# A prospective analysis of lymphocyte phenotype and function over the course of acute sepsis

**DOI:** 10.1186/cc11404

**Published:** 2012-06-28

**Authors:** Jonathan S Boomer, Jennifer Shuherk-Shaffer, Richard S Hotchkiss, Jonathan M Green

**Affiliations:** 1 Department of Internal Medicine, Washington University School of Medicine, 660 S. Euclid Ave, St Louis, MO 63110, USA; 2 Departments of Anesthesia and Surgery, Washington University School of Medicine, 660 S. Euclid Ave, St Louis, MO 63110, USA

## Abstract

**Introduction:**

Severe sepsis is characterized by an initial hyper-inflammatory response that may progress to an immune-suppressed state associated with increased susceptibility to nosocomial infection. Analysis of samples obtained from patients who died of sepsis has identified expression of specific inhibitory receptors expressed on lymphocytes that are associated with cell exhaustion. The objective of this study was to prospectively determine the pattern of expression of these receptors and immune cell function in patients with acute sepsis.

**Methods:**

Twenty-four patients with severe sepsis were enrolled within 24 hours of the onset of sepsis, as were 12 age-matched healthy controls. Peripheral blood was obtained at enrollment and again seven days later. Immune cell subsets and receptor expression were extensively characterized by quantitative flow cytometry. Lymphocyte function was assayed by stimulated cytokine secretion and proliferation assays. Results were also correlated to clinical outcome.

**Results:**

At the onset of severe sepsis, patients had decreased circulating innate and adaptive immune cells and elevated lymphocyte expression of receptors associated with cell activation compared to controls. Samples analyzed seven days later demonstrated increased expression of the inhibitory receptors CTLA4, TIM-3 and LAG-3 on T lymphocytes accompanied by decreased expression of the IL-7 receptor. Functional assays revealed impaired secretion of interferon γ following stimulation *in vitro*, which was reversible by incubation overnight in fresh media. Impaired secretion of IFNγ correlated with death or development of secondary infection.

**Conclusions:**

Lymphocytes from patients with acute sepsis upregulate expression of receptors associated with cell exhaustion, which may contribute to the immune suppressed state that occurs in protracted disease. Therapy that reverses T cell exhaustion may restore immune function in immunocompromised patients and improve survival in sepsis.

## Introduction

Sepsis is characterized by an intense systemic response to infection in which patients typically present with marked respiratory and hemodynamic instability [1]. The initial phase of sepsis is thought in large part to be result of a 'cytokine storm' caused by the activation of innate and adaptive immune cells and the systemic release of pro-inflammatory mediators [2,3]. While some patients rapidly recover, others have a more protracted course characterized by multiple organ dysfunction syndrome (MODS). Many patients with sepsis develop secondary bacterial infections and these may be caused by strains that are relatively non-pathogenic in normal hosts [4]. In addition, patients with sepsis frequently reactivate latent viruses such as herpes simplex virus (HSV) or cytomegalovirus (CMV) [5,6]. These observations have suggested that a subset of individuals with sepsis enter a more immune suppressed state.

During acute sepsis, the release of pro-inflammatory cytokines, such as IL-1β and IL-6, and the immune modulatory cytokine, IL-10, by innate immune cells such as macrophages, granulocytes and natural killer (NK) cells has been well documented (reviewed in [2]). This initial phase appears to be followed by a rapid induction of apoptosis of both innate and adaptive immune cells in a caspase-dependent manner [7-10]. Furthermore, a consistent decrease in HLA-DR expression, an essential molecule for antigen presentation, and expression of co-stimulatory molecules such as CD86 has also been observed [11-13]. This initial phase of activation and apoptosis may be accompanied by increased numbers of suppressor cells, such as regulatory T cells (Treg), myeloid derived suppressor cells (MDSCs) and the recently described CD11b+/CD62L+ population of granulocytes, as a mechanism for controlling the adaptive immune response and returning the body to homeostasis [14-19].

We recently published an analysis of tissues obtained by rapid bedside autopsy from a series of patients who died as a consequence of sepsis and found a cellular phenotype consistent with immune exhaustion [20]. This phenotype was originally described in the mouse lymphochoriomeningitis virus (LCMV) model and has been subsequently identified in chronic viral infections in humans including HIV and chronic hepatitis C infection [21-24]. Exhausted T cells fail to secrete cytokines, have reduced proliferation in response to antigen and express certain cell surface receptors (that is, TIM-3, LAG-3, CD69, cytotoxic T lymphocyte antigen-4 (CTLA-4) and PD-1) while also decreasing the expression of the IL-7R on their cell surface [25,26]. Experimental data suggest that T cell exhaustion may be reversible by interfering with signaling through inhibitory receptors such as PD-1 [24,27-30]. Thus, if this is an important mechanism of immune-suppression in sepsis, there may be opportunities to intervene therapeutically.

By virtue of the study design, there were several important limitations of the post-mortem study. Only those patients who died during the course of their illness were included, consequently we were unable to determine if the phenotype was present in all patients with sepsis or only in those who succumbed from the disease. As samples were obtained at only a single time point (death) we were unable to determine changes that occurred during the course of illness. Furthermore, it is possible that the phenotype was present at the outset or even prior to sepsis, and in fact only identifies those already immune-suppressed and therefore at higher risk of death should they become septic.

To address these questions and determine which, if any, of our findings might be present in living patients with sepsis, we performed a prospective observational trial. Patients admitted to the intensive care unit within 24 hours of the onset of severe sepsis were identified and enrolled. Peripheral blood was obtained at the start of the study (day 0) and then again at day seven. Immune cells were isolated from the blood and extensively characterized for their expression of activating and inhibitory receptors as well as for immune function following *in vitro *stimulation. Data were then correlated to clinical outcome, namely development of a secondary infection or death. Control samples were obtained from age- and sex-matched healthy volunteers to determine the normal variance in expression patterns of these receptors. We determined that in comparison to control subjects, there were differences in immune cell populations in the blood and that over time, patients with sepsis had increased expression of specific inhibitory receptors and ligands on T cells and antigen presenting cells. In addition, we identified both immune cell phenotypes and functions that correlated with the development of secondary infection and/or death.

## Materials and methods

### Patients and controls

Patients older than 18 years of age admitted to the medical or surgical ICU at Barnes-Jewish Hospital with a diagnosis of sepsis were identified. Severe sepsis was defined using a consensus panel definition of the presence of systemic inflammatory response syndrome (SIRS) criteria, known or suspected source of infection and organ failure [31]. All patients had respiratory failure requiring mechanical ventilation on enrollment. Patients with active malignancy, HIV infection, chronic Hepatitis B or C infection or on immunosuppressive medications (except corticosteroids at a dose of < 10 mg prednisone or equivalent per day) were excluded. Consent was obtained from the patients legally authorized representative, as all patients enrolled were judged too acutely ill to provide valid consent. Onset of severe sepsis, defined by the time at which the subject met consensus criteria for severe sepsis was confirmed by the study physician to be within 24 hours of enrollment. Control subjects consisted of normal age-matched volunteers with no significant concomitant acute or chronic illnesses. Clinical data on the septic patients was captured daily. Patients were monitored daily for evidence of new infection, and considered to have developed a secondary infection if there were new positive cultures and/or clinical data such as new fever, elevated white blood cells (WBC) and new infiltrate on chest X-ray consistent with new infection. All protocols were approved by the Washington University Institutional Review Board.

### Sample collection and processing

Heparinized blood was collected through an indwelling central venous catheter, arterial line or venipuncture (septic patients) or by peripheral venipuncture (controls) on day 0 and again at the end of the study (day seven unless otherwise indicated). The blood was immediately transported and processed in the research laboratory. Peripheral blood mononuclear cells (PBMC) were isolated by ficoll-hypaque density gradient separation, using standard protocols. Plasma was collected and stored at -80°C for cytokine determinations. The cells were washed and resuspended in T cell media (Roswell Park Memorial Institute (RPMI) 1640 supplemented with 10% heat-inactivated FCS, hydroxyethyl piperazineethanesulfonic acid (HEPES), penicillin/streptomycin, L-glutamine and non-essential amino acids) and processed for staining, proliferation or cytokine secretion as described below.

### Flow Cytometry

All antibodies were purchased from BD Biosciences (San Diego, CA, USA), eBiosciences (San Diego, CA, USA) or Biolegend (San Diego, CA, USA). For surface marker staining 100 μl of whole blood was incubated with 10 μl human AB serum and the indicated fluorescently conjugated antibodies for one hour at room temperature followed by hypotonic red blood cell lysis. The cells were extensively washed in PBS containing 1% BSA and then resuspended in PBS containing 1% BSA and 1% paraformaldehyde (PFA). For intracellular staining of FoxP3, following surface staining with α-CD4 and α-CD25, cells were permeabilized with Fix/Perm buffer (eBiosciences) and incubated with either control or α-FoxP3 antibody for one hour at 4°C, washed then resuspended in PBS/BSA containing 1% PFA. Viable lymphocytes were identified by forward scatter (FSC) and side scatter (SSC) properties and exclusion of 7-aminoactinomycin D (7-AAD). T cells were identified as either CD4+ or CD8+. Regulatory T cells were identified as CD4+ and CD25+ that co-labeled with FoxP3. NK cells were identified as CD56+ while natural killer T (NKT) cells co-labeled with CD3 and CD8+ NK cells co-labeled with CD8. Dendritic cells were identified as lineage cocktail negative (CD3/CD19/CD16/CD14/CD20/CD56), HLA-DR+ and sub-typed as plasmacytoid dendritic cells (pDC, CD123+) or monocytoid dendritic cells (mDC, CD11c+). MDSC were identified as lineage cocktail negative, HLA-DR low/negative, CD33+ and CD11b+. All samples were analyzed by flow cytometry on a FACSCalibur cytometer (Becton Dickinson Corp., Mountainview, CA, USA). Data were further analyzed using WinList Software (Verity Corporation, Topsham, ME, USA).

### Proliferation and cytokine analysis

PBMCs were resuspended in T cell media at 2 × 10^6 ^cells/ml and 50 μl added to each well of a round bottom 96-well tissue culture plate. The cells were either left unstimulated or treated with α-CD3 (1 μg/ml, OKT3, eBiosciences) in combination with α-CD28 (1 μg/ml, clone CD28.2, eBiosciences) or with phorbol myristate acetate (PMA) (5 ng/ml) plus ionmycin (0.4 μg/ml). After 48 hours, 1 μCi of tritiated thymidine (^3^H-TdR) was added to each well and allowed to incubate overnight. Cells were harvested onto glass microfiber filters and counts of incorporated ^3^H-TdR determined by liquid scintillography. For determination of cytokine production, cells were cultured as described for proliferation and culture supernatant obtained at five hours and 48 hours following stimulation. Levels of cytokine present in plasma and culture supernatants were determined using the cytokine bead array (BD Biosciences) according to the manufacturer's protocol using the human Th1/Th2/Th17 kit that measures IL-2, IL-4, IL-6, IL-10, IL-17A, TNF-α and IFNγ. For some samples, the cells were allowed to rest overnight in fresh media, then stimulated as above and culture supernatant collected after five hours for cytokine determinations.

### Data analysis and statistics

To objectively quantify the per cell expression level of each receptor, fluorescein isothiocyanate (FITC) and phycoerythrin (PE). Quantum MESF beads (Bangs Laboratories, Fishers, IN, USA) were run with each flow cytometric aassay. The Quantum beads are microspheres, each of a fixed fluorescence, which when run on a flow cytometer provide individual peaks (six peaks for FITC and five peaks for PE) of fluorescence. These peaks of known fluorescence intensities are converted to Molecules of Equivalent Soluble Fluorochrome (MESF) units, using a proprietary spreadsheet provided by Bang Laboratories, Inc., generating a standard curve. The mean fluorescence intensity (MFI) of each marker is than converted to MESF units based on the Quantum Bead MESF standard curve. As the Quantum MESF beads are run on the flow cytometer at the same time as the subject samples using the same instrument and settings, samples obtained from different subjects and/or at different times can be directly compared. Statistical significance was determined as a *P *value < 0.05 upon either a two-tailed non-parametric Wilcoxon matched pairs test or a two-tailed Mann Whitney U test (Prism4, GraphPad Software, Inc. La Jolla CA).

## Results

### Patient enrollment and specimen collection

Admissions to the medical and surgical ICU were screened daily for potential subjects. Those who met enrollment criteria were identified and their legally authorized representative approached to obtain informed consent. Twenty-four subjects were enrolled and initial samples collected. Of these 24 subjects, second samples were collected from 16 at day seven while five subjects died prior to the collection of a second sample. One subject had the second sample collected on day nine and two subjects had samples collected immediately prior to the withdrawal of life-sustaining therapies (on day two and day five). The clinical characteristics of the septic population are shown in Table 1. As expected, the patients were acutely ill with a median Acute Physiology and Chronic Health Evaluation II (APACHE II) score of 20 and a mortality of just over 40%. A number of comorbidities were present as is typical for this patient population. Three subjects were on low dose corticosteroids at the time of enrollment. Control subjects were identified and selected to approximately age- and sex-match the patient population. Only those with no underlying chronic health conditions were included as controls.

**Table 1:** Clinical characteristics of patients with sepsis and controls.

Parameters	Patients with sepsis (*n *= 24)	Control subjects (*n *= 12)
Age, median years (range)	62 (29 to 91)	57 (44 to 59)
Males, percentage (number)	54 (13)	42 (5)
APACHEII at admission, median (range)	20 (10 to 33)	n/a
MOD score at admission, median (range)	6 (1 to 10)	n/a
28-day mortality, percentage (number)	42 (10)	n/a
Nosocomial infection, percentage (number)	21 (5)	n/a
Length of ICU stay, median days (range)	6.5 (1 to 28)	n/a
Length of hospitalization, median days (range)	11.5 (1 to 89)	n/a
Ventilation days, median days (range)	4 (1 to 19)	n/a
WBC at enrollment, median cells/μL × 10^-3 ^(range)	12.8 (0.4 to 47)	n/a
Absolute lymphocyte count, median cells/μl × 10^-3 ^(range)	0.97 (.07 to 4.8)	n/a
Comorbidities, percentages		none
End stage renal disease	17	
Cirrhosis	17	
Diabetes	13	
Cardiovascular disease	35	

APACHEII, Acute Physiology and Chronic Health Evaluation II; MOD, multiple organ disfunction; n, number; WBC, white blood cell

### Characterization of circulating immune cells at onset of severe sepsis

**Figure 1:**
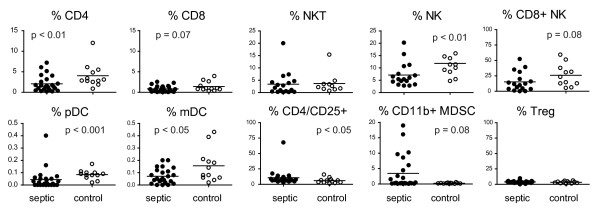
**Characterization of immune cell subsets at the onset of sepsis**. Peripheral blood was collected from patients within 24 hours of the onset of severe sepsis and from controls and analyzed by flow cytometry to determine the percentage of circulating cells of each subset. The markers used to identify each subset are described in the methods. Horizontal lines indicate the mean value. *P *values were calculated by a non-parametric 2-tailedMann-Whitney U-test.

**Figure 2:**
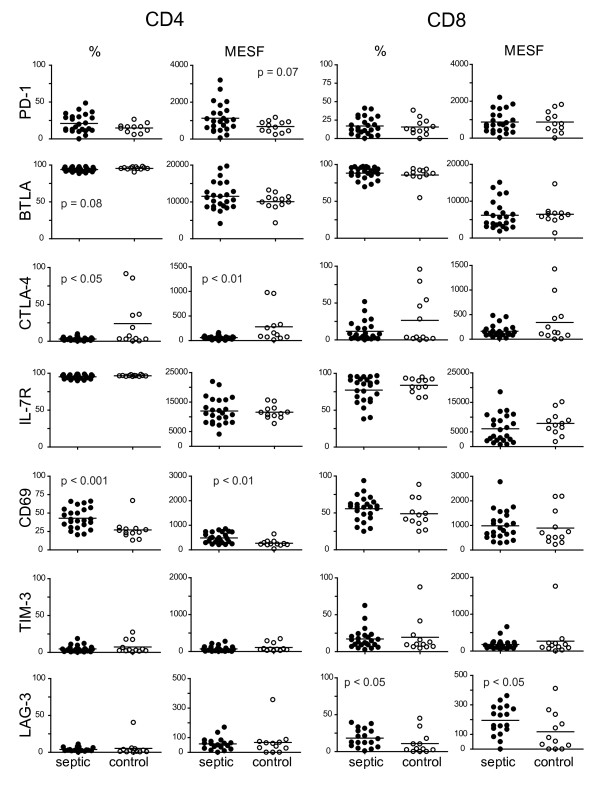
**Determination of expression pattern of specific receptors on CD4- and CD8-positive T cells at the onset of severe sepsis**. Peripheral blood was collected from patients within 24 hours of the onset of severe sepsis and from normal controls and stained for the indicated markers. The percent positive relative to isotype control staining was determined. Fluorescence intensity was quantified by calculation of MESF values as described in the methods. Horizontal lines indicate the mean value. *P *values were calculated by a non-parametric 2-tailedMann-Whitney U-test. MESF, molecules of equivalent soluble fluorochrome.

At enrollment the median total WBC count was 12,900 cell/μl with an absolute lymphocyte count (ALC) of 971 cells/μl. Half of the subjects were lymphopenic with an ALC of < 1,000. Over the first week, the ALC increased on average by 340 cells/μl, but this change was not statistically significant. To determine the distribution of lymphocyte subsets as well as expression of cell surface molecules on specific cell populations, blood was obtained within 24 hours of the onset of severe sepsis and extensively characterized by flow cytometry (Figures 1 and 2). An identical analysis was performed on healthy control subjects to provide a comparison population. The markers used to identify each cell population are described in the methods. As shown in Figure 1, there was a reduction in the percentage of CD4+ T cells and a trend towards a decrease in CD8+ T cells in the blood of acutely septic patients. A corresponding decrease in innate immune cells was also observed in the blood of septic patients with the percentage of NK cells and dendritic cells (both pDC and mDC) being reduced. Expression of CD86, a ligand for the T cell costimulatory receptor CD28, was decreased on mDC. While there was no difference in the percentage of Treg cells at day 0, there was a trend towards an increase in CD11b+ myeloid derived suppressor cells, a population implicated in mediating immune suppression in cancer and infection [18,19] in acutely septic subjects.

In the acute phase of sepsis, both lymphocyte activation and depletion has been documented [2,3]. T cells isolated from the blood of acutely septic individuals also had elevated expression of CD25, as a percentage of CD4+ T cells (Figure 1), and of CD69 (Figure 2), as both a percentage of positive cells and expression level per CD4+ T cell (MESF), indicative of an activated phenotype. Interestingly, we also observed a trend in elevated expression, as determined by MESF (*P *= 0.07), of Programmed Death Receptor-1 (PD-1), an important negative regulator of lymphocyte function, in the septic individuals compared to healthy subjects. The other major inhibitory receptors expressed by T cells are BTLA and CTLA-4. The majority of T cells expressed BTLA (> 90%) in both septic and non-septic individuals. There was a higher percentage of CD8+ T cells that expressed LAG-3 as well as elevated per cell expression when compared to age-matched healthy individuals. No other significant differences in CD8+ T cell expression of either activation or inhibitory receptors were observed. These data suggest that the observed changes are specific and not due to non-specific upregulation of all cell surface receptors.

### Changes in receptor expression over the course of acute sepsis

**Figure 3:**
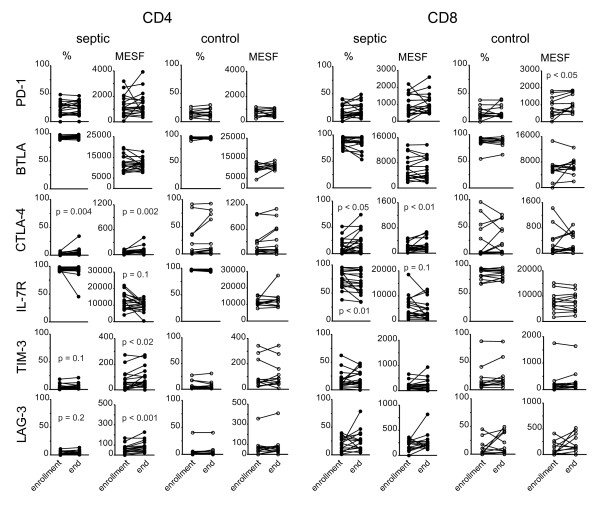
**Changes in receptor expression on CD4 and CD8 T cells over the course of acute sepsis**. Peripheral blood was collected at enrollment and again seven days later from patients with severe sepsis and from normal controls and analyzed by flow cytometry for the indicated markers on CD4- and CD8-positive T cells. The lines connect values at enrollment and the end of the study from an individual subject. Percentages and MESF values were calculated as described in the methods. *P *values were calculated by 2-tailed, non-parametric Wilcoxin matched pairs test. MESF, molecules of equivalent soluble fluorochrome.

**Figure 4:**
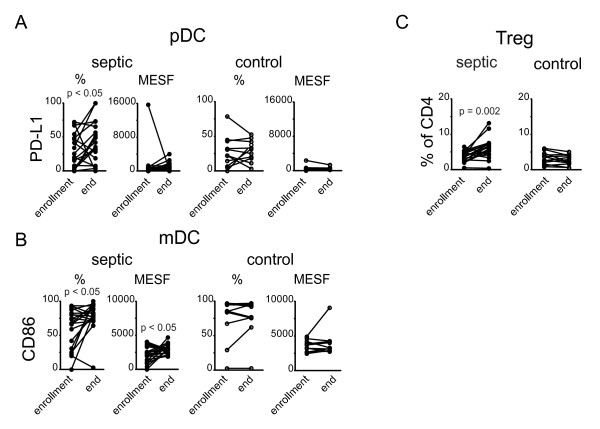
**Changes in expression of inhibitory and costimulatory ligands on dendritic cell subsets and regulatory T cell percentages over the course of acute sepsis**. Peripheral blood was collected at enrollment and again seven days later from patients with severe sepsis and from normal controls and analyzed by flow cytometry for the presence of regulatory T cells (Treg) and dendritic cells (DC). The plasmacytoid and myeloid DC subsets were analyzed for expression of the inhibitory ligand PD-L1 or costimulatory ligand CD86 respectively. The lines connect values at enrollment and the end of study from an individual subject. Percentage and MESF values were calculated as described. *P *values were calculated by 2-tailed, non-parametric Wilcoxin matched pairs test. MESF, molecules of equivalent soluble fluorochrome.

We hypothesized that specific changes in receptor expression patterns over the course of acute sepsis would be detected. In particular, we were interested in whether receptor ligand pairs that had been implicated in inhibiting lymphocyte function were upregulated over time. Therefore, we repeated the flow cytometric analysis on samples obtained later in the disease. Except as noted in the methods, the repeat analysis was performed on day seven for all subjects in whom a second sample was able to be collected. As presented in Figure 3, changes in the expression of several receptors were noted over this time interval. On both CD4+ and CD8+ T cells, we observed a trend towards a decrease in per cell expression of the IL-7 receptor (CD127) and a decrease in the overall percentage of CD8+ cells that expressed the IL-7R. Expression of TIM-3 and LAG-3, as determined by quantitative MESF values was also increased on CD4+ T cells. On CD4+ and CD8+ T cells, we detected an increase in expression of CTLA-4 by MESF as well as an increase in the percentage of cells expressing CTLA-4. Models of cell exhaustion have reported persistent elevated expression of the activation marker CD69. We found that patients with sepsis did have a higher expression of CD69 on CD4+ T cells at the onset of sepsis, as well as a higher circulating percentage of CD69 expressing CD4+ T cells and this did not significantly change over the course of the acute illness (data not shown). The percentage of pDC that expressed the ligand for PD-1, PD-L1, increased over the duration of sepsis, as did expression of CD86 on mDC (Figure 4). Although we did not detect any difference in the percentage of Treg cells at the onset of sepsis, this increased significantly over the interval in septic patients but not in normal controls (Figure 4). This pattern of increased inhibitory receptor expression is consistent with our findings in patients who died of sepsis, and is compatible with the phenotype of immune cell exhaustion. Smaller, but statistically significant changes in the expression of a few markers were also detected in control subjects, highlighting the need for caution in interpreting these results.

**Figure 5:**
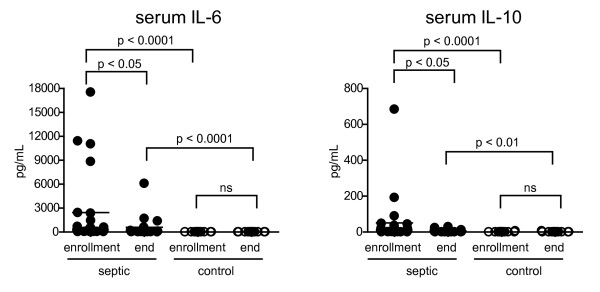
**Plasma cytokine levels over the course of acute sepsis**. Plasma obtained at enrollment or at the end of study participation was collected from patients with severe sepsis and from normal controls. Cytokine levels were determined by the Cytokine Bead Array. Horizontal lines indicate the mean value. *P *values were calculated by a non-parametric 2-tailed Mann-Whitney U-test.

We measured the level of a panel of seven circulating cytokines in the plasma of patients with sepsis and controls. Of those measured, only IL-6 and IL-10 were different with the remaining cytokines being very low or undetectable in either subject population (Figure 5 and Additional File 1, Table S1). Both IL-6 and IL-10 were significantly higher in patients with sepsis and both decreased markedly from enrollment to the end of the study. Three of the four patients with the highest IL-6 levels at enrollment died, two during the first week and one later in the hospitalization. We did not detect any relationship of IL-10 levels or the IL-6:IL-10 ratio to clinical outcome (data not shown).

### Stimulated PBMC cytokine secretion and proliferation in patients with sepsis

**Figure 6:**
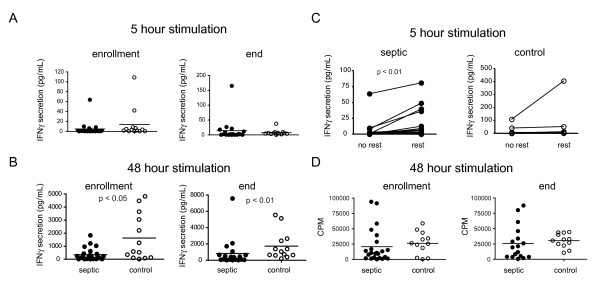
**Functional characterization of peripheral blood mononuclear cells from patients with sepsis**. Peripheral blood was collected at enrollment and again seven days later from patients with severe sepsis and from normal controls. Peripheral blood mononuclear cells (PBMCs) were isolated and stimulated with α-CD3 and α-CD28 antibodies for five hours (panel **A**) or 48 hours (panel **B**) and analyzed for IFNγ secretion. A duplicate plate of PBMCs was rested overnight in fresh media prior to stimulation with α-CD3 and α-CD28 antibodies (panel **C**). Proliferation was determined by tritiated thymidine incorporation on PBMCs obtained at enrollment and at the end of study participation and stimulated for 48 hours with α-CD3 and α-CD28 antibodies (panel **D**). *P *values were calculated by a non-parametric 2-tailed Mann Whitney U-test (panels A and B) or a 2-tailed paired Students T-test (panel C).

We had previously reported that PBMCs isolated from patients who died of sepsis had a profound global reduction in cytokine secretion when stimulated *in vitro *[20]. To determine whether the defect was present in patients with acute sepsis, we performed a similar analysis on PBMCs isolated at enrollment and at the end of the study. In contrast to our findings with post-mortem specimens, in this study we found that cytokine secretion from PBMCs activated *in vitro *was largely preserved with the notable exception of IFNγ (Figure 6 and Additional File 2, Table S2). Secretion of IFNγ following 48 hours of stimulation with α-CD3/α-CD28 antibody was markedly reduced compared to normal controls in samples obtained at either enrollment or at the end of study participation.

One potential explanation for the observed defect in IFNγ secretion was that factors present or absent in the septic milieu might reversibly impair cytokine secretion. To test this, the cells were either stimulated immediately or rested overnight in fresh media and then stimulated for five hours with α-CD3/α-CD28. As shown in Figure 6C, the rested cells from patients with sepsis, but not controls, had significantly greater secretion of IFNγ (rested: 16.1 ± 23.1 pg/ml versus no rest: 5.7 ± 15.7 pg/ml *P *< 0.01). The fact that overnight rest in fresh media restored IFNγ secretion to levels near those of controls subjects suggests that the impairment may in fact be reversible, perhaps by removal of an inhibitory factor or addition of a growth factor present in fresh media.

As an additional measure of lymphocyte function, we assessed the proliferative capacity of PBMCs following *in vitro *stimulation (Figure 6D). The mean proliferation of PBMCs stimulated with α-CD3/α-CD28 from septic individuals was similar to non-septic individuals at enrollment and at the end of the study. Therefore, although there was a decrease in IFNγ secretion, there is not a global impairment in all cellular functions in this patient population.

### Plasma IL-6 and stimulated IFNγ secretion correlate with clinical outcome

**Figure 7:**
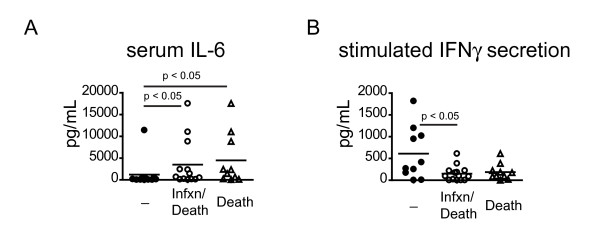
**Elevated plasma IL-6 and impaired stimulated IFNγ secretion are correlated with outcome**. At the time of enrollment, plasma was collected and PBMCs were isolated and stimulated for 48 hours with α-CD3 and α-CD28 antibodies. Plasma IL-6 levels (**A**) or PBMC stimulated IFNγ secretion (**B**) was measured by Cytokine Bead Array. The levels were compared between patients who recovered uneventfully and those who died or who either died or developed a second infection. *P *values were determined by a non-parametric 2-tailed Mann Whitney U-test.

We were interested in determining which, if any, of the immunologic variables measured correlated with a clinically significant outcome. All patients with sepsis were followed over the course of their hospitalization for overall mortality or evidence of a new infection. In addition, we determined if patients were re-admitted within 28 days of the enrollment with a new infection. In the course of the study ten patients died as a result of their illness, six patients developed a second infection (two of whom died. Two infections occurred after discharge and resulted in readmission within 28 days.) and ten patients survived without a second infection. We analyzed whether any parameters we measured at enrollment correlated with either second infection and mortality together or mortality alone. Consistent with previous reports, patients who died or had second infections had higher plasma IL-6 levels than those who had an uneventful recovery (Figure 7A) [32]. Interestingly, those patients who developed a second infection or died had significantly less IFNγ secretion from *in vitro *stimulated PBMCs than those who recovered uneventfully (Figure 7B). These data are supportive of the hypothesis that functional immune cell impairment may predispose to a poor outcome.

## Discussion

The acute phase of sepsis is characterized immunologically by activation of the innate immune cells, resulting in an intense pro-inflammatory state and 'cytokine storm'. The initial pro-inflammatory state is countered by a compensatory anti-inflammatory response, which if excessive or prolonged may result in a significant element of immune suppression [3,33]. Thus, sepsis results in the activation of essentially all facets of the immune system. Understanding the temporal changes that occur in immune cells during the course of sepsis is critical for determining when the immune response has become more harmful than helpful, and for designing rational therapeutic strategies to intervene when necessary.

We had previously extensively characterized the immune cells isolated from the spleens and lungs of patients who had succumbed to severe sepsis [20]. The results suggested profound immunosuppression in this population of patients, as evidenced by a failure of lymphocytes to secrete cytokines after *in vitro *stimulation as well as a pattern of elevated cell surface expression of inhibitory receptors and ligands. However, the post-mortem study was limited in only analyzing cells obtained at the time of death. By virtue of its design, that study could not address whether the phenotype observed was present at initiation of sepsis or developed over the course of the disease. In the current study we performed a prospective analysis of patients with acute sepsis. Immune cell phenotype and function were extensively characterized at the onset of sepsis and then again one week later. Our findings confirm several important results of the post-mortem study, and provide additional insights into immune system function during sepsis. One limitation of this study is that we only examined a limited number of patients, thus our results require validation on a larger sample size. A further limitation of this current study is that our control population consisted only of normal healthy individuals and not critically ill non-septic patients; therefore, any findings in the septic patient population may be due to acute critical illness or perhaps even due to underlying co-morbid disease and not necessarily from sepsis *per se*. However, an important aspect of this study is the temporal change in immune status. In this analysis the comparison is to the same subject at time 0, therefore they serve as their own control. Thus, independent of the lack of a critically ill control population, our findings document the status of the immune system at the outset of sepsis and the temporal changes that occur during the disease.

Our findings demonstrated a reduction in circulating innate (NK and DC) and adaptive (CD4+) immune cells in samples obtained at enrollment, consistent with reports demonstrating significant lymphopenia and cell death early in the course of disease [7-10,34-39]. While cell death is important, the evolution of the phenotype and functional status of the remaining cells over the course of the disease may also be a determinant of the host response and outcome. Many studies have examined only limited cell populations and receptors and/or at only a single time point. A strong association of monocyte HLA-DR expression with mortality and development of nosocomial infection in patients with sepsis has been found, demonstrating the clinical utility and importance of understanding the status of the immune system during sepsis [12,40,41]. In the current study, we included an extensive battery of markers to provide a detailed picture of the lymphocyte phenotype at the onset and again at a later stage of sepsis. An important caveat to the interpretation of data from many previous studies is the lack of a reproducible quantitative methodology for determining the expression levels of cell surface proteins, limiting the ability to compare specimens obtained and analyzed at different times in a controlled manner, as discussed in a recent review by Venet *et al. *[42]. By incorporating the use of reference fluorescent beads with each analysis, we were able to standardize fluorescence measurements to a standard curve and accurately compare measurements between subjects and time points.

In early sepsis, most markers we examined were not different from normal controls, with the exception of increased expression of the early activation marker CD69 and the IL-2 receptor (CD25) on CD4+ T cells and the inhibitory receptor LAG-3 on CD8+ T cells as well as a small but statistically significant reduction in CTLA-4 expression. PD-1 expression on CD4+ T cells was somewhat higher than in controls, but did not quite reach statistical significance (*P *= 0.07). Over time, we found the expression of the inhibitory receptor CTLA-4 on both CD4+ and CD8+ T cells and TIM-3 and LAG-3 on CD4+ T cells increased on cells from septic patients. Both CD4+ and CD8+ T cells had decreases in expression of the receptor for the homeostatic cytokine IL-7 over the course of the study. We did observe minor changes in receptor expression patterns in the control patients; however, these tended to be smaller in magnitude than those observed in the septic patients and driven by only a few subjects. Nonetheless, this does suggest that there are normal variations within an individual over time that must be accounted for in any analysis of this type.

The change in receptor expression pattern that we observed over the course of sepsis, increased TIM-3, LAG-3 and CD69 with decreased IL-7R expression, is consistent with the phenotype of immune cell exhaustion, although the functional impairment was not as severe. Best characterized in murine systems, there is a growing body of evidence that the exhausted phenomenon is important in human disease, including chronic Hepatitis B, Hepatitis C and HIV infection [24,26,30,43-46]. In HIV, exhausted T cells display elevated levels of PD-1, TIM-3, LAG-3 and CTLA-4 and treatment with a TIM-3 blocking antibody restored T cell function [47]. In Hepatitis B and C, interference with PD-1 restored T cell responses [24,45]. These data illustrate the importance of understanding the expression of regulatory receptors on immune cells and the potential for manipulation of these pathways. Future studies examining the expression and function of these inhibitory receptors in patients with sepsis, non-septic critically ill and other control populations will be of particular interest.

In addition to changes of receptor expression in the lymphocyte compartment, myeloid and plasmacytoid dendritic cells also displayed temporal changes in the expression of ligands for costimulatory and inhibitory receptors. Increased expression of PD-L1 on pDC was accompanied by an increase in CD86 and a decrease in CD80 on mDC over the course of the study (Figure 4 and data not shown). These changes are consistent with murine studies demonstrating an important role for CD80 and CD86 in the response to sepsis, and with a reciprocal pattern of regulation of these receptors on circulating monocytes in humans with sepsis [13,48].

The observation that there is overlapping expression of activating receptors and ligands on T cells and antigen presenting cells (APCs) is consistent with a model of immune cell exhaustion occurring over a continuum. Early in the immune response activating signals predominate, but also induce expression of inhibitory receptors. In the face of ongoing antigen stimulation, there may be downregulation of activating receptor:ligand pairs with persistent expression of inhibitory receptors and ligands. The temporal sequence may differ depending on many factors including the cell type examined and the nature of the ongoing stimulus. Thus, depending on when sampled, the actual expression pattern may be predominantly one of activating receptors, inhibitory receptors or both [26,49,50].

Recently, there have been several reports suggesting an important role for PD-1 in sepsis [51-54]. PD-1 is a receptor expressed on activated lymphocytes, which when engaged by its ligands PD-L1/PD-L2, inhibits cell proliferation and function [55]. In both animal models and humans, PD-1 has been implicated in the immune suppression that is often observed in malignancy and in chronic viral infections. In particular, experimental evidence supports an important role for PD-1 signaling in contributing to immune cell exhaustion characteristic of certain chronic viral infections [27,28,56,57]. In experimental murine sepsis, blockade of PD-1 or PD-L1 improved survival and in humans upregulated PD-1 expression has been detected and correlated with worse outcome [52,53]. In our study, PD-1 expression on CD4+ T cells was slightly higher than controls at day one, and although the increase in expression by day seven was not significant, it was higher than day seven controls, both in the percentage of PD-1 expressing CD4+ T cells (24% versus 15%, *P *< 0.05) and the MESF (1,413 versus 682, *P *< 0.005). Concurrently, we detected an increase in the percentage of pDC that expressed PD-L1, a ligand for PD-1, from enrollment to the end of the study in septic patients. Thus, our data provide further evidence in support of a role for the PD-1/PD-L axis in sepsis.

Suppressor cell populations have been shown to be increased in septic individuals [14,17,19,58-61]. These may be an important mechanism to down-modulate the inflammatory response but also carry the potential for deleterious consequences. Although we did not observe an increase in the percentage of Tregs at day 0 relative to controls (Figure 4), the percentage increased over the course of the disease. However, whether this is due to an expansion of the Treg population or a loss of CD4+CD25- cells, as has been described, cannot be definitively determined [62]. Myeloid derived suppressor cells, were noted to be increased at 24 hours in septic patients, although the level did not quite reach statistical significance (*P *= 0.08) and proceeded to return to baseline levels by day seven in our septic subjects (data not shown). This provides another potential mechanism whereby innate cells can inhibit T cell responses early in the course of the disease until the control systems of the adaptive immune system (that is, Tregs) become functional.

Our functional analysis of the immune cells revealed a relatively selective defect in the secretion of IFNγ, with preserved secretion of other cytokines. While the impaired IFNγ secretion was consistent with our previous findings, the preservation of other cytokines is counter. One explanation for this discrepancy may be that many of these subjects are fundamentally different than those who died. Death from sepsis is the most extreme outcome, and it is likely that the organ dysfunction, including immune cell impairment, is greater in those who die. In addition, in the post-mortem analysis, both splenic and lung tissue from septic patients had aberrant expression of the ligands for inhibitory receptors on antigen presenting and parenchymal cells [20]. The fact that in the current study we did not observe as severe an impairment of function in circulating cells suggests that an important mechanism of immunosuppression may in fact be local inhibition of lymphocyte function by engagement of inhibitory receptors by ligand expressed in the peripheral tissue.

We did observe that the most severe impairment of IFNγ secretion at enrollment was correlated with either development of secondary infection or death. Thus, this may be an important marker to identify those at risk. Furthermore, the reversibility of the impairment in immune cell function may be an important factor in the ability of subjects to recover and in developing therapeutic approaches to augment immune cell function. Our data suggest that the impaired function, as measured by IFNγ secretion, may be reversible, as cells first rested overnight in fresh media had greater cytokine secretion than cells stimulated immediately on isolation from the blood. Identification of the factor(s) responsible for either inhibiting or augmenting T cell function may provide important mechanistic insights.

As a further assessment of lymphocyte function, we measured proliferation in response to stimulation with α-CD3 and α-CD28. The proliferative capacity was not decreased relative to controls in specimens obtained either at enrollment or at the end of the study. Previously published work has demonstrated reduced proliferation in samples obtained from patients with sepsis [52]. In that study samples were isolated at day three to five of sepsis and stimulated with the non-specific mitogen, phytohemagglutinin (PHA), whereas we examined cells either at day one or day seven and activated with α-CD3/α-CD28 which more selectively induces T cell proliferation. These experimental differences, as well as other methodological differences may account for the discrepancy.

## Conclusions

Acute sepsis is characterized by the loss of circulating innate and adaptive immune cells and impaired secretion of IFNγ. Over the first week of illness, patients with sepsis upregulate the expression of a number of important inhibitory receptors and their ligands on the surface of lymphocytes and dendritic cells, downregulate expression of the receptor for IL-7 and increase the numbers of Treg cells. Combined, these changes are consistent with an early stage of immune cell exhaustion and may be important in predisposing some patients to nosocomial infection or poor outcome, as well as suggest further targets for development of treatment intervention strategies.

## Key messages

• At the onset of sepsis, while there is evidence of loss of innate and adaptive immune cells, there are only minor differences in the expression of inhibitory receptors on circulating lymphocytes.

• Over the first week of acute sepsis, lymphocytes upregulate specific inhibitory receptors and downregulate the IL-7 receptor, a phenotype associated with cell exhaustion. Thus, the exhausted phenotype is not present at the earliest stage of sepsis, but may develop during the course of the disease.

• Lymphocytes from patients with sepsis have impaired secretion of IFNγ when measured at both the onset of sepsis and again later in the course of the disease. This is reversed by overnight rest of the cells in fresh media prior to stimulation.

• Impaired IFNγ secretion correlated with clinically significant morbidity and mortality.

## Abbreviations

7-AAD: 7-aminoactinomycin D; ALC: absolute lymphocyte count; APACHE II: Acute Physiology and Chronic Health Evaluation II; BSA: bovine serum albumin; BTLA: B and T lymphocyte attenuator; CBA: cytokine bead array; CMV: cytomegalovirus; CTLA-4: cytotoxic T lymphocyte antigen-4; FCS: fetal calf serum; HCV: hepatitis C virus; HSV: herpes simplex virus; ^3^H-TdR: tritiated thymidine; HVEM: herpes viral entry mediator; IFN: interferon; IL: interleukin; LAG-3: lymphocyte-activation gene 3; LCMV: lymphochoriomeningitis virus; mDC: myeloid dendritic cell; MDSC: myeloid derived suppressor cell; MESF: molecules of equivalent soluble fluorochrome units; MFI: mean fluorescence intensity; MODS: multiple organ dysfunction syndrome; NK: natural killer cells; NKT: natural killer T cells; PBMC: peripheral blood mononuclear cells; PBS: phosphate buffered saline; PD-1: programmed cell death receptor -1; pDC: plasmayctoid dendritic cell; PD-L: programmed death receptor ligand; PFA: paraformaldehyde; PHA: phytohemagglutinin; PMA: phorbol myristate acetate; SIRS: systemic inflammatory response syndrome; TIM-3: T cell immunoglobulin mucin-3; TNF: tumor necrosis factor; Treg: regulatory T cells; WBC: white blood cell count.

## Competing interests

The authors declare that they have no financial or non-financial competing interests.

## Authors' contributions

JSS screened and enrolled subjects as well as performed most of the assays reported in this paper. JSB optimized many of the assays, interpreted the data, prepared figures and wrote sections of the paper. RSH contributed to the experimental design and interpretation. JMG designed the study, oversaw all aspects of the study, interpreted the data and prepared final drafts of all figures as well as wrote the manuscript. All authors reviewed and approved of the final paper.

## Supplementary Material

Additional file 1 **Supplemental Table 1: Plasma cytokine levels**. Plasma was collected at study enrollment and again at the end of the protocol. Cytokines were measured by multiplex ELISA (Cytokine Bead Array, BD Biosciences). Values are reported as pg/ml. The mean ± standard deviation are shown. *P *values were calculated using a non-parametric 2-tailed Mann-Whitney U-test. Click here for file

Additional file 2 **Supplemental Table 2: Stimulated PBMC cytokine secretion**. PBMCs were isolated from patients with sepsis or normal controls at enrollment and again at the end of the protocol. The cells were stimulated *in vitro *with α-CD3 and α-CD28 antibodies for either five or 48 hours and culture supernatants analyzed for cytokine content by multiplex ELISA (Cytokine Bead Array, B-D Biosciences). Results shown are mean ± standard deviation for all subjects and reported as pg/ml. * = *P *< 0.05. ** = *P *< 0.01 by Mann-Whitney test. Click here for file
