# Supplementary material for: A prospective analysis of lymphocyte phenotype and function over the course of acute sepsis
Source: Crit Care. 2012 Jun 28;16(3):R112. doi: 10.1186/cc11404 (PMC3580670; doi:10.1186/cc11404)
Supplement: Additional file 1 — Supplemental Table 1: Plasma cytokine levels. Plasma was collected at study enrollment and again at the end of the protocol. Cytokines were measured by multiplex ELISA (Cytokine Bead Array, BD Biosciences). Values are reported as pg/ml. The mean ± standard deviation are shown. P values were calculated using a non-parametric 2-tailed Mann-Whitney U-test. [file cc11404-S1.DOC]

Supplemental Table 1: Plasma cytokine levels (pg/ml)

| **Septic** | | | **Controls** | | |
| --- | --- | --- | --- | --- | --- |
|  | **enrollment** | **end** | **enrollment** | **end** | **p value** |
| **IL-2** | 0 | 0.4±1.8 | 0 | 0 |  |
| **IL-4** | 0 | 0 | 0 | 0 |  |
| **IL-6** | 2445.0±4703***** | 588.3±1454**^** | 0.5±1.3***** | 0.6±1.6**^** | *****≤.0001, **^**≤.0001 |
| **IL-10** | 50.8±141.1***** | 5.0±8.4**^** | 0.6±1.8***** | 0.5±1.8**^** | *****≤.0001, **^**≤.001 |
| **IL-17A** | 0.1±0.6 | 3.5±7.7 | 0 | 0 |  |
| **TNF** | 0 | 0 | 0 | 0 |  |
| **IFN-γ** | 0 | 1.3±4.7 | 0 | 0 |  |

Plasma was collected at study enrollment and again at the end of the protocol. Cytokines were measured by multiplex ELISA (Cytokine Bead Array, BD Biosciences). P values were calculated using a non-parametric 2-tailed Mann-Whitney U-test.
