# Supplementary material for: A prospective analysis of lymphocyte phenotype and function over the course of acute sepsis
Source: Crit Care. 2012 Jun 28;16(3):R112. doi: 10.1186/cc11404 (PMC3580670; doi:10.1186/cc11404)
Supplement: Additional file 2 — Supplemental Table 2: Stimulated PBMC cytokine secretion. PBMCs were isolated from patients with sepsis or normal controls at enrollment and again at the end of the protocol. The cells were stimulated in vitro with α-CD3 and α-CD28 antibodies for either five or 48 hours and culture supernatants analyzed for cytokine content by multiplex ELISA (Cytokine Bead Array, B-D Biosciences). Results shown are mean ± standard deviation for all subjects and reported as pg/ml. * = P < 0.05. ** = P < 0.01 by Mann-Whitney test. [file cc11404-S2.DOCX]

Supplemental Table 2: Stimulated PBMC cytokine secretion (pg/ml)

| enrollment | | | | |
| --- | --- | --- | --- | --- |
|  | **septic** | | **control** | |
|  | *5 hour* | *48 hour* | *5 hour* | *48 hour* |
| IL-2 | 41.8±100.7 | 319.5±807.3 | 47.2±94.6 | 119.0±133.2 |
| IL-4 | 1.9±4.8 | 13.7±27.5 | 2.7±7.0 | 18.5±30.7 |
| IL-6 | 4.5±8.5 | 100.9±146.5 | 8.2±14.7 | 292.3±409.0 |
| IL-10 | 0.8±2.0 | 74.0±120.4 | 2.5±6.2 | 217.1±456.0 |
| IL-17A | 2.0±7.9 | 51.6±171.2 | 5.9±14.5 | 56.3±139.7 |
| TNFα | 66.0±125.7 | 649.3±952.3 | 85.2±136.7 | 749.2±912.0 |
| IFN-γ | 4.9±14.1 | 342.3±462.6***** | 13.8±30.7 | 1623.0±1786.0***** |

| end | | | | |
| --- | --- | --- | --- | --- |
|  | **septic** | | **control** | |
|  | *5 hour* | *48 hour* | *5 hour* | *48 hour* |
| IL-2 | 67.7±111.5 | 232.5±371.7 | 29.4±47.3 | 168.5±226.4 |
| IL-4 | 2.5±7.6 | 15.0±24.6 | 1.0±2.7 | 15.3±16.3 |
| IL-6 | 36.3±104.2 | 457.2±1264.0 | 9.9±20.0 | 305.3±540.7 |
| IL-10 | 1.7±3.6 | 113.1±208.9 | 0.7±1.5 | 82.5±97.6 |
| IL-17A | 6.8±17.9 | 133.4±277.3 | 0.3±1.2 | 13.9±22.8 |
| TNFα | 132.2±214.1 | 895.6±1261.0 | 67.7±73.2 | 635.5±602.6 |
| IFN-γ | 14.4±38.4 | 826.8±1731.0** | 7.3±9.4 | 1735.0±1752.0****** |

PBMCs were isolated from patients with sepsis or normal controls at enrollment and again at the end of the protocol. The cells were stimulated *in vitro* with α-CD3 and α-CD28 antibodies for either 5 or 48 hours and culture supernatants analysed for cytokine content by multiplex ELISA (Cytokine Bead Array, B-D Biosciences). Results shown are mean ± standard deviation for all subjects. * = P < 0.05. ** = P < 0.01 by Mann-Whitney test.
